# Nutritional screening in surgical patients of a teaching hospital from Southern Brazil: the impact of nutritional risk in clinical outcomes

**DOI:** 10.1590/S1679-45082013000200002

**Published:** 2013

**Authors:** Rosane Scussel Garcia, Léa Regina da Cunha Tavares, Carla Alberici Pastore

**Affiliations:** 1 Universidade Federal de Pelotas, Pelotas, RS, Brazil; 2 Hospital Escola da Fundação de Apoio Universitário, Universidade Federal de Pelotas, Pelotas, RS, Brazil

**Keywords:** Nutritional therapy, Malnutrition, Surgical procedures

## Abstract

**Objective:**

To assess the prevalence of nutritional risk in surgical patients of a teaching hospital and its associated factors.

**Methods:**

A cross-sectional study with secondary data of surgical ward patients of the *Hospital Escola da Universidade Federal de Pelotas*, from April to October, 2010. Patients were evaluated up to 36 hours after admission using the Malnutrition Screening Tool.

**Results:**

The study included 565 patients, with a mean age of 52.8±15.6 years, and the majority (51%) was female. More than 30% of the patients presented with an average or high nutritional risk, and 7% of them were at high risk. Associated with the greater risk were aging, cancer surgery, and mortality. The length of hospital stay showed a linear increase according to nutritional risk.

**Conclusion:**

The Malnutrition Screening Tool is a simple and effective tool for nutritional screening that does not require anthropometric measurements. In this study, average or high nutritional risk was prevalent in one third of the sample, and was related to increased mortality, hospital stay, cancer, and aging. Nutritional care outpatients’ protocols could be used prior to elective surgery to reduce the nutritional risk of these patients, improving clinical outcomes and reducing length and costs of hospital stay.

## RESUMO

**Objetivo:**

Avaliar a prevalência de risco nutricional em pacientes cirúrgicos de um hospital universitário e seus fatores associados.

**Métodos:**

Estudo transversal com dados secundários de pacientes da ala cirúrgica do Hospital Escola da Universidade Federal de Pelotas, de abril a outubro de 2010. Os pacientes foram avaliados até 36 horas após a internação, sendo aplicado o *Malnutrition Screening Tool*.

**Resultados:**

Foram incluídos 565 pacientes, com idade média de 52,8±15,6 anos, sendo a maioria (51%) do gênero feminino. Mais de 30% dos pacientes apresentaram risco nutricional médio ou alto, sendo cerca de 7% com alto risco. Estiveram associados ao maior risco nutricional: avanço da idade, cirurgias neoplásicas e mortalidade. O tempo de permanência hospitalar mostrou aumento linear de acordo com o risco nutricional.

**Conclusão:**

O *Malnutrition Screening Tool* é uma ferramenta simples e efetiva de rastreamento nutricional, dispensando medidas antropométricas. Neste estudo, médio ou alto risco nutricional foi prevalente em um terço da amostra e esteve relacionado com aumento de mortalidade, de permanência hospitalar, câncer e aumento da idade. Protocolos de cuidados nutricionais ambulatoriais prévios a cirurgias eletivas poderiam ser empregados na redução do risco nutricional desses pacientes, melhorando os desfechos clínicos e reduzindo o tempo e os custos da internação.

## INTRODUCTION

Intrahospital malnutrition is an important public health problem, since it increases morbidity and mortality of patients who do not present with an adequate nutritional status at hospital admission.

Malnutrition proves to be associated with longer hospital stay^(1-3)^, and higher rates of complications^(4)^ and mortality^(5)^, also implying higher hospital costs. The cost of hospitalization increases by approximately 68% in malnourished patients, due to longer hospital stays, greater expenses with medications to treat complications (especially infectious), as well as greater costs of nutritional support to treat the established malnutrition^(6)^.

A large study called the *Inquérito Brasileiro de Nutrição* (IBRANUTRI, acronym in Portuguese) [The Brazilian Nutrition Survey] that sought to delineate the nutritional profile of inpatients in various hospitals all over Brazil, revealed that well-nourished patients stay, in average, six days at hospital, while those moderately malnourished have an average stay of 9 days and the severely malnourished individuals, an average of 13 days. Increased hospital stay corresponds to increased risk of infections and complications, as well as increased costs, since besides prolonged occupation of the bed, there is an increase in costs with medications and treatments necessary to care for these complications^(2)^. The same study demonstrated that about 48% of the hospitalized population in Brazil has some degree of malnutrition^(2)^.

Even in developed countries, intrahospital rates of malnutrition are high, such as in England (20%)^(3)^ and in Australia (36%)^(5)^. In developing countries, such as in Latin American, the prevalence of malnutrition in hospitalized patients is about 50%^(7,8)^.

Despite being prevalent, malnutrition is frequently not recognized and is undertreated in clinical practice^(9)^. Some studies showed that less than 50% of malnourished patients received appropriate nutritional treatment since their nutritional status was not effectively recognized^(10,11^).

In order to improve the approach to and identification of patients with nutritional risk, i.e., those who are more likely to have malnutrition during their hospital stay, the routine use of simple screening procedures is recommended^(11)^.

Nutritional screening identifies individuals who are malnourished or at risk of developing malnutrition, and those who can benefit from specific nutritional support. For this, it is necessary to apply a simple, effective, and validated tool for hospital use^(12)^.

Several tools were developed for nutritional screening, such as the Malnutrition Universal Screening Tool (MUST)^(13)^, Imperial Nutritional Screening System (INSYST)^(14)^, Short Nutritional Assessment Questionnaire (SNAQ)^(15)^, Mini Nutritional Assessment (MNA)^(16)^– for elderly patients –, the *Avaliação Subjetiva Global* (ASG) [Global Subjective Evaluation]^(17)^ – which can be used either as a screening tool or for assessing nutritional status –, and the Malnutrition Screening Tool (MST)^(12)^.

The MST is a quick, simple, validated, and effective tool, and it is plausibly applied to the large volume of patients who are daily admitted to hospitals^(12)^. It is a sensitive screening method for detecting patients at nutritional risk, and takes 3 to 5 minutes to be completed and interpreted^(18)^.

The MST has the ease of not requiring the patient's weight and height, data that is not always present during the first hours of hospitalization, often due to the impossibility of moving the patient. Focusing on subjective data, such as history of weight loss and loss of appetite, and changes in the patient's food ingestion, this tool identifies patients at risk, and shows a good correlation with other methods that are lengthier, more complex, and that need anthropometric data of the individual, such as weight and height^(18)^.

Three studies carried out at the same hospital in London (United Kingdom), during the years 1998, 2000 and 2003, showed that from the time when the hospital adopted the nutritional screening process, the prevalence of malnutrition was significantly reduced (from 23.5% in 1998, to 20.4% in 2000, and to 19.1% in 2003). This reduction is attributed to screening, since it allows identification and treatment of patients with nutritional risk before they, in fact, become malnourished^(19)^.

## OBJECTIVE

To evaluate the prevalence of nutritional risk in hospitalized patients and the length of stay required by such patients, according to their category of nutritional risk at the time of admission.

## METHODS

This is a cross-sectional study conducted in patients hospitalized in the surgical ward of the *Hospital Escola da Universidade Federal de Pelotas* (RS), from April to October, 2010, using secondary data taken from admission medical charts of these patients (history taken and screening already routinely performed by the hospital's Nutrition Service). This hospital serves exclusively through the Public Brazilian Unified Healthcare System (SUS).

Excluded from the study were patients aged under 18 years or those whose information, for whatever reason, could not be reliably collected.

The patients admitted were evaluated by the nutrition team up to 36 hours after admission. Nutrition students, under the supervision of hospital dieticians, applied the questionnaires and the routine assessments for the nutritional procedures of the service.

One of the standardized questionnaires at the service is the MST^(12)^, the tool chosen by the service for nutritional screening due to quickness and ease of completion, with no need for anthropometric data at the beginning of the hospital stay, and that can be applied by any duly trained healthcare professional. The MST covers three issues: if there was recent non-intentional weight loss; if yes, how great the loss was; and if the patient has been eating poorly due to less appetite. These questions generate a numerical score, in which 0-1 point indicate a low risk and a need for reevaluation (performed every 7 days at the service, if the patient is hospitalized), 2-3 points indicate an average risk, and 4-5 points indicate high risk; patients at average or high risk undergo a detailed history taking, nutritional evaluation, and diet therapy.

After collecting data from the medical charts (gender, age, type of surgery, and MST result) and outcome for the patient (discharged, transferred, or death), the questionnaires were entered into a Microsoft Excel^®^ databank, with double entry and checking for consistency. The convenience sample was collected for 7 months, reaching 565 patients. Statistical analyses were made using the Stata 9.1^®^ package, and values of p<0.05 were accepted as significant.

This survey did not involve exposure of the patient to any type of risk to his/her health or personal exposure. The data collected were secondary, thus exempting them from signing the participants' Informed Consent Form.

This project was approved by the Research Ethics Committee responsible for the *Hospital Escola da Universidade Federal de Pelotas*, as per Official Notice # 45/10, of October 4, 2010.

## RESULTS

The study included 565 patients, most of them (51%) females. The mean age of the sample was 52.8 years (±15.6 years), varying from 18 to 91 years. Most patients had as outcome hospital discharge (96.6%). The complete description of the sample may be found on table 1.

**Table 1:** Description of sample of surgical patients at the *Hospital Escola da Universidade Federal de Pelotas*, RS, 2010

Variable		n	%
Gender			
	Male	275	48.7
	Female	290	51.3
Age			
	<45 years	166	29.4
	45-60 years	194	34.3
	>60 years	205	36.3
Outcome			
	Discharge	546	96.6
	Death	11	2.0
	Transfer	8	1.4
Total		565	100

The mean time of hospitalization was 7.4 days (±10.0 days), with a maximum of 89 days. This variable did not show a normal distribution (Skewness/Kurtosis normality test – p=0.000), so that, in terms of results, this variable is presented in the form of median and interquartile interval (IQI).

The operations performed were of various types, most of them related to neoplasms (38.6%), and followed by biliary lithiasis surgery, as is shown on table 2.

**Table 2:** Surgical diagnoses in the sample of patients at the *Hospital Escola da Universidade Federal de Pelotas*, RS, 2010

Surgery	n	%
Neoplasm head/neck	24	4.2
Neoplasm – respiratory system	50	8.8
Neoplasm – gastrointestinal tract	77	13.6
Other neoplasms*	67	11.9
Biliary lithiasis (cholecystectomy / videocholecystectomy)	137	24.3
Herniorraphy	78	13.8
Other surgeries**	132	23.4
Total	565	100

* Other neoplasms include neoplasm of genitourinary tract, prostate, soft tissues, lower and upper limbs;

** Other surgeries include hemorrhoidectomy, thyroidectomy, non-oncological surgeries of the urinary, digestive and respiratory tracts; and new surgeries due to non-oncological postoperative complications.

The results obtained from the application of the MST show that most patients did not present with weight loss prior to admission (57%) or any change in food intake (74%). The nutritional screening tool showed that 33.1% of the patients in the surgical ward of the hospital presented with an average or high nutritional risk, and almost 7% were at high risk (Table 3). Among the patients, 18% had lost between 1 and 5kg, and almost 11% had lost more than 10kg relative to their habitual weight.

**Table 3:** Results of applying the Malnutrition Screening Tool to the sample of surgical patients at the *Hospital Escola da Universidade Federal de Pelotas*, RS, 2010

MST variable		n	%
Recent weight loss			
	No	321	56.8
	Does not know	15	2.7
	1-5kg	102	18.1
	6-10kg	59	10.4
	11-15kg	33	5.8
	>15kg	27	4.8
	Yes, do not know how much	8	1.4
Decreased habitual diet			
	No	416	73.6
	Yes	149	26.4
Nutritional risk classification			
	Low risk (0-1 point)	378	66.9
	Medium risk (2-3 points)	148	26.2
	High risk (4-5 points)	39	6.9
Total		565	100

MST: Malnutrition Screening Tool.

**Figure 1:**
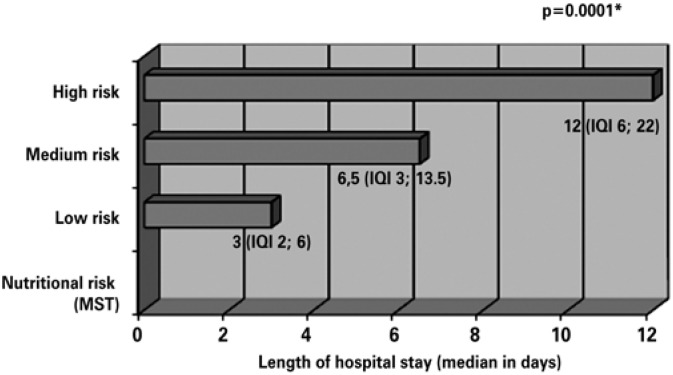
Relation between nutritional risk and length of hospital stay in a sample of surgical patients at the *Hospital Escola da Universidade Federal de Pelotas*, RS, 2010

The nutritional risk was not significantly different between genders (p>0.05, χ^2^ test), but there were differences related to age, surgical diagnosis, outcome of patients (Table 4), and length of hospital stay (Figure 1).

**Table 4:** Factors associated to nutritional risk in a sample of surgical patients at the *Hospital Escola da Universidade Federal de Pelotas*, RS, 2010

Associated variable		Nutritional risk						p value*
		Low		Medium		High		
		n	%	n	%	n	%	
Age (years)								<0.001
	<45	129	34.1	34	23.0	3	7.7	
	45-60	134	35.5	45	30.4	15	38.5	
	>60	115	30.4	69	46.6	21	53.8	
Diagnosis								<0.001
	Neoplasm	116	30.7	79	53.4	23	59.0	
	Others	262	69.3	69	46.6	16	41.0	
Outcome								<0.001
	Discharge	376	99.5	138	93.2	32	82.1	
	Transfer	2	0.5	4	2.7	2	5.1	
	Death	0	0.0	6	4.1	5	12.8	
Total		378	100	148	100	39	100	

* χ^2^ test.

As to age, the higher the age group of patients, the greater the prevalence of high nutritional risk, reaching 54% in patients over 60 years of age (p<0.001, χ^2^ test). The surgical diagnosis of neoplasm was related to a greater nutritional risk when compared to the other types of surgery; cancer patients had a high nutritional risk (59%) *versus* those with other diagnoses (41%). Only 31% of the oncology patients showed a low nutritional risk, while in diagnoses different from cancer, this value increased to 69% (p<0.001, χ^2^ test).

When the hospital outcome of patients is observed, the rise in mortality rates according to increased nutritional risk is remarkable: zero per cent in patients with a low risk, to 4% in those with a medium risk, and reaching the level of 13% in patients with a high nutritional risk (p<0.001, χ^2^ test). Having a high nutritional risk brought with it a probability three times higher of death relative to patients with a medium nutritional risk (prevalence ratio of 3.16, with a 95% confidence interval (95% CI) of 1.02-9.82; p=0.04).

Time of hospital stay showed a linear increase according to the increased nutritional risk. The length of hospital stay of high-nutritional-risk patients was four-fold longer than that of low-risk patients, achieving a median of 12 days, while patients with a medium risk had a median stay of 6.5 days (p<0.001, Kruskal-Wallis test).

## DISCUSSION

Recurrent failure in recognizing and treating malnutrition, especially where it is very frequent (hospital), is an obstacle to healthcare. Under these conditions, the routine use of a simple screening, capable of identifying the patient's nutritional risk, is recommendable^(11)^.

Nutritional screening benefits the individuals who are malnourished or at a risk for malnutrition who may be benefited from nutritional support^(12)^.

In the present study, the screening done with the MST^(12)^ resulted in one third of the patients with nutritional risk, in which the majority of these were identified with medium risk. Many studies measured the prevalence of nutritional risk in hospitalized patients, using diverse tools for this. The MST, however, has not been frequently used in the studies published, since most have used the ASG as a screening tool upon hospital admission^(17)^.

Studies using ASG reported a prevalence of malnutrition/nutritional risk in inpatients (clinical and surgical) between 36 and 50%^(5,7,8)^, in different countries of the world. A study using anthropometrics and body mass index to define malnutrition found a prevalence of 20% in a general hospital population^(3)^. Lamb et al., using the MST nutritional screening tool^(13)^, found 44% of nutritional risk – in that, 13.7% patients were at a high risk. In a literature review on nutritional screening, Elia et al.^(11)^ reported a prevalence of hospital nutritional risk between 10 and 60%, depending on the tool used, and the present study corroborates the data of that review.

In 2008, Vidal et al.^(1)^ published an article in which, using ASG as the tool, they found 40.2% of prevalence of malnutrition among surgical and clinical patients at a Spanish hospital, showing that there was no difference in terms of prevalence between the two types of patients. This same study pointed out that 54% of the surgical patients had decreased their habitual food intake, while in the present study only about one fourth of the patients reported this reduction.

In the study entitled IBRANUTRI^(2)^, with hospitalized patients in general (not only surgical) evaluated by ASG, Waitzberg et al. showed a malnutrition prevalence of 48.1%, in which 12.5% were severe cases of malnutrition. It is important to point out that IBRANUTRI assessed patients in many hospitals from north to south of the extensive Brazilian territory, and found large regional discrepancies. In the north of the country, a region with the lowest income *per capita*, the prevalence of malnutrition reached 78.8%. In the south of the country, the prevalence was 38.9%, close to the prevalence reported in the present study, which was conducted at a hospital in the extreme southern region of Brazil. Despite the use of distinct tools, the regional results are similar.

The increase in patient age was significantly associated with the higher nutritional risk in the sample studied here. Also in the studies by Waitzberg et al.^(2)^, Middleton et al.^(5)^, Correia et al.^(7)^, Wyszynski et al.^(8)^, and Lamb et al.^(10)^, even using distinct screening tools, the increase in age was related to the increase in nutritional risk/malnutrition, primarily for those over 60 years of age.

Diagnosis of the patient was also associated with the nutritional risk in the present study. Finding similarity with several other studies, the diagnosis of cancer was related to a greater nutritional risk^(1,2,7,8)^. In the IBRANUTRI^(2)^ study, patients with cancer presented with a risk 55% higher of being malnourished than did non-cancer patients.

In the sample studied, mortality was linearly greater as the nutritional risk increased. In a study carried out in Sydney, Australia, Middleton et al.^(5)^ described a 12-month mortality after evaluation with ASG almost three-fold higher among malnourished patients relative to those who were well nourished. Regarding deaths during hospitalization, 2.7% of the malnourished patients died, while only 1% of the well-nourished patients had this outcome.

In the present study, the increase in nutritional risk was significantly associated with length of hospital stay (p<0.001). Similar findings were described in other studies^(1,2,5,7)^. In Brazil, the IBRANUTRI^(2)^ study reports a median hospital stay of 6 days for well-nourished patients, 9 days for moderately malnourished/with risk of malnutrition, and 13 days for severely malnourished patients. In a multicenter study conducted in several Latin American countries, Correia et al.^(7)^ found a relative risk of 3.00 (95% CI: 2.61-3.45) of malnourished patients having a hospital stay of more than 14 days relative to the well-nourished.

MST is a simple, quick, valid, and safe tool that can be used to identify patients with nutritional risk^(12)^. It has the ease of not using anthropometric data for screening, which makes it feasible during the first hours of hospital admission, even if the patient cannot move for measuring weight and height.

In their article, Edington et al.^(3)^ reported a loss of 15.9% of their eligible population (1611 patients), since 256 individuals could not be weighed. The MST, with questions about recent weight loss and loss of appetite, can be applied without anthropometric measurements, and has convergent and predictive validity relative to other nutritional screening methods^(12)^.

## CONCLUSION

The MST proved to be a simple and effective tool for nutritional screening with the advantage of exempting the patient from anthropometric measurements, not always available during the first hours of hospital admission.

In this study, greater nutritional risk was related to an increase in mortality, length of hospital stay, diagnosis of cancer, and increased age.

There is substantial evidence that treatable malnutrition is under-recognized and undertreated. There are formal recommendations from societies of nutritional therapy from around the world and Brazil that patients admitted to hospitals be screened and that the screening process be repeated periodically. In this way, patients that can benefit from nutritional support will be identified in a correct and early manner.
